# Artificial intelligence-based algorithm for predicting outcomes in early-stage lung cancer: An annotation-free imaging artificial intelligence study

**DOI:** 10.1016/j.xjon.2026.101742

**Published:** 2026-03-30

**Authors:** Keiju Aokage, Jumpei Ukita, Mamoru Miura, Keisuke Ogaki, Tomoko Kataoka, Noriko Mitome, Mitsuhiro Isaka, Masaya Yotsukura, Shun-ichi Watanabe, Masahiro Tsuboi

**Affiliations:** a Department of Thoracic Surgery, National Cancer Center Hospital East, Chiba, Japan; b M3 Inc, Tokyo, Japan; c Japan Clinical Oncology Group Data Center/Operations Office, National Cancer Center Hospital, Tokyo, Japan; d Division of Thoracic Surgery, Shizuoka Cancer Center, Shizuoka, Japan; e Department of Thoracic Surgery, National Cancer Center, Tokyo, Japan

**Keywords:** artificial intelligence, annotation-freee, thin-section computed tomography, non–small cell lung cancer, surgery, personalized care

## Abstract

**Objective:**

Surgery remains the standard treatment for clinical stage I non–small cell lung cancer (NSCLC). Conventional prognostic factors are often subjective and variable, highlighting the need for objective prediction. We developed and validated an annotation-free artificial intelligence (AI) model using computed tomography (CT) images and clinical data to predict prognosis in stage I NSCLC.

**Methods:**

In step 1, an AI algorithm was developed to predict pathological classifications from CT and clinical data collected in 3 prospective multi-institutional trials. In step 2, the model was refined and validated using a cohort from the National Cancer Center Hospital East. Models were trained to predict 5-year disease-free survival and overall survival. Performance was evaluated by sensitivity, specificity, predictive values, and receiver operating characteristic area under curve (AUC).

**Results:**

We analyzed 1217 patients in step 1 and 1338 in step 2. Models integrating CT imaging and clinical data outperformed models using either dataset alone. The pathology prediction model achieved an AUC of 0.787. The highest performance for 5-year disease-free survival (AUC = 0.757) and overall survival (AUC = 0.756) was obtained by combining preoperative clinical information, physician CT assessments, and an AI-based CT model. Adding AI outputs to clinical factors improved risk stratification, better separating high-risk from low- to intermediate-risk groups.

**Conclusions:**

Annotation-free AI models that integrate CT imaging with clinical data provide accurate, objective prediction of recurrence and survival in stage I NSCLC, complement conventional diagnostics, and support personalized multidisciplinary treatment planning.

**Figure:**
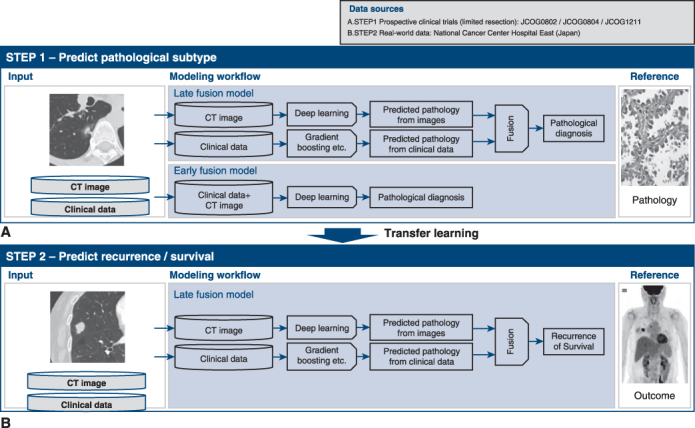
AI model integrating images and data to predict pathology and survival outcomes.

**Central Message**

A novel AI algorithm without an annotation of lung nodules predicts recurrence and survival in stage I NSCLC, offering transformative potential for personalized surgeryand preoperative care.

**Perspective**

This study introduces a cutting-edge, annotation-free artificial intelligence algorithm that does not require tumor segmentation, integrating preoperative CT imaging and clinical data to predict recurrence and survival in stage I NSCLC. This automated approach enables personalized surgical strategies and proactive perioperative management, improving outcomes and decision making.

Surgery is the standard of care for treating clinical stage I non–small cell lung cancer (NSCLC). Currently, the prognostic classification of clinical stage I lung cancer is based on the T factor, which is primarily determined based on the maximum solid-component diameter. According to the International Association for the Study of Lung Cancer, the 5-year overall survival (OS) rates for clinical stages IA and IB disease are 82% and 69%, respectively.^1^ The tumor characteristics observed on chest computed tomography (CT) play crucial roles in selecting surgical procedures and estimating prognosis. Predicting pathological invasion and prognosis in patients with clinical stage I NSCLC is crucial for optimizing treatment strategies (eg, surgery and adjuvant chemotherapy) that are considered based on pathological stage. The Japan Clinical Oncology Group (JCOG) 0201 observational study, which was aimed at predicting invasive lung cancer via CT imaging, identified the consolidation-to-tumor ratio as the most reliable predictor of pathological tumor invasion.^2^ Additionally, various other predictors, including the solid diameter on thin-section CT,^3^, ^4^, ^5^ maximum standardized uptake value observed in positron emission tomography images,^6^ tumor markers,^7^^,^^8^ pleural indentation, and air bronchogram have been reported. However, conventional predictors have limitations, including subjectivity and variability in interpretation. With advancements in artificial intelligence (AI) and its expanding applications in medicine, AI-based diagnostic tools potentially represent more objective and universally standardized diagnostic methods. The development of an AI-based prognostic model for stage I lung cancer could facilitate more precise risk stratification, leading to the development of improved surgical strategies and perioperative treatment approaches. In this study, we aimed to leverage AI-based deep-learning technology to improve pretreatment prognosis estimation in patients with clinical stage I NSCLC and contribute to the development of optimal treatment strategies.

## Methods

### Participants and Study Design

This study was conducted in 2 sequential steps. In step 1, we analyzed the data of patients eligible for 3 clinical trials (JCOG0804, JCOG0802, and JCOG1211), focusing on function-preserving surgery for clinical stage I lung cancer and available preoperative CT imaging data.^9^, ^10^, ^11^ These trials, conducted as multicenter trials within the JCOG, constituted cohort 1.

**Figure 1:**
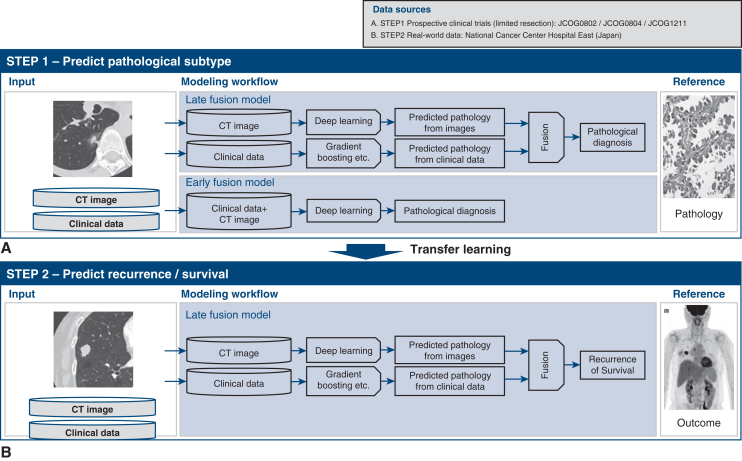
Clinical setup. *JCOG*, Japan Clinical Oncology Group; *CT*, computed tomography.

In step 2, the machine-learning prediction model developed in step 1 was further refined and validated using external data. Cohort 2 included 1366 patients with clinical stage 0 to IB lung cancer who underwent surgery at the National Cancer Center Hospital East between January 2003 and December 2014. A survival and recurrence prediction algorithm were constructed using these datasets, incorporating a model subjected to transfer learning from the pathology-prediction AI algorithm developed in step 1 (Figure 1). This study was registered with University Hospital Medical Information Network on May 20, 2025 (UMIN000057915).

### Step 1

In step 1, we developed a model for predicting the pathological classification using imaging and clinical data from cohort 1. Regarding pathological classification as the outcome variable, all patients in cohort 1 were diagnosed according to the Noguchi classification; however, pathological classification based on the fourth edition of the World Health Organization (WHO) classification was available only for JCOG1211.^12^^,^^13^ This discrepancy arose due to differences in the timing of clinical trials. To address this issue, the data were transformed into each classification, and several models were created and analyzed. Noguchi and colleagues^12^ proposed a histopathological classification for small lung adenocarcinomas (≤2 cm), categorizing lesions into 6 types (A-F) and stratifying prognosis based on tumor structure, scar formation, and the extent of invasion. Noguchi types A and B represent noninvasive lesions characterized predominantly by a lepidic growth pattern and are associated with an excellent prognosis. Noguchi type C is defined as minimally invasive carcinoma with a central scar and still shows a favorable prognosis. In contrast, Noguchi types D through F demonstrate definite invasion and are therefore regarded as invasive adenocarcinomas. In the 2015 and 2021 WHO classifications, lung adenocarcinomas are classified according to the presence of invasion and the invasive size into adenocarcinoma in situ (AIS), minimally invasive adenocarcinoma (MIA), and invasive adenocarcinoma.^13^ This system closely corresponds to the Noguchi classification: Noguchi types A and B generally correspond to AIS, type C to MIA, and types D through F to invasive adenocarcinoma. Disease-specific survival for AIS and MIA has been reported to be nearly 100%, which is consistent with the prognostic stratification of the Noguchi classification. Therefore, we defined the outcome variable according to the correspondence table shown in Online Data Supplement.

Two previously established methods, namely late-fusion and early-fusion models, were compared as potential approaches for integrating various types of data, including imaging and clinical information (Figure 1).^14^ With the late-fusion model, CT images were analyzed using deep learning to establish a convolutional neural network-based model that estimates the probability of each pathological classification.^15^ Additionally, a supervised machine-learning model was developed using a gradient-boosting method incorporating clinical data to estimate the probability of each pathological classification.^16^^,^^17^ The outputs of these models were then merged to calculate the final probability of each pathological classification. In contrast, the early-fusion model integrated CT images and clinical data as inputs into a machine-learning algorithm, which then applied deep learning to predict the probability of each pathological classification.

For both models, all data were exclusively divided into training and evaluation datasets in a 3:1 ratio, ensuring a patient-wise split to prevent data leakage. The models were trained using the training data, and their performances were verified using the evaluation data. The performance metrics included sensitivity, specificity, positive-predictive value, negative-predictive value, and receiver operating characteristic-area under the curve (ROC-AUC) data.

In addition, the classification of each tissue was evaluated using a confusion matrix and the quadratic-weighted Kappa index.^18^ These metrics were used to assess whether the pathological diagnosis could classify invasive tissue subtypes, defined as Noguchi classification types D through F and invasive adenocarcinoma (excluding lepidic adenocarcinoma) according to WHO classifications. Additional evaluations were performed in terms of Noguchi classification type C, lepidic adenocarcinoma, type B, and minimally invasive carcinoma.

When CT images were used as input, we used a combined model involving CT image preprocessing^19^ and extraction of the region of interest (ROI) of the tumor area,^20^ following previous research.^21^ However, because region of interest (ROI) extraction yielded no difference in accuracy compared with an end-to-end deep learning approach that uses the entire CT image as input, we did not perform ROI extraction. We employed 3D ResNet-18^22^ architecture as the backbone network, wherein this architecture was pretrained on the Huazhong University of Science and Technology lung CT dataset (for pretraining) dataset.^23^ Furthermore, the pathological classifications were used to generate the output of each model by matching the WHO classification and the Noguchi classification, factoring in the pathological findings.

### Step 2

In step 2, a new model was constructed to predict the probability of recurrence using preoperative information (including tumor marker carcinoembryonic antigen, age, sex, and smoking history) and CT findings assessed by a physician (including maximum tumor diameter, presence of ground-glass opacity, and consolidation-to-tumor ratio) from cohort 2, using an annotation-free CT-imaging data learned from cohort 1 for pretraining. The aim of this step was to assess whether the prognosis and recurrence could be predicted based on imaging and clinical data. The inclusion criteria were as follows: patients with age at surgery aged 80 years or younger, clinical stage of 0 through IB, performance status of 0 or 1, pathologically confirmed complete resection, and available preoperative thin-section CT data. Patients with missing clinical data, unavailable or nonstandardized Digital Imaging and Communications in Medicine images, or images lacking both lungs were excluded from the analysis.

In the late-fusion model,^14^ a deep learning-based model^15^ was first constructed to predict pathological classifications using only CT images from cohort 1 as a pretrained model. Then, the entire model was retrained with CT images from cohort 2 to estimate 5-year OS and 5-year disease-free survival (DFS) rates. For the 5-year binary disease-free survival and OS end points, time 0 was the date of surgery and the outcome was classified as event within 5 years if recurrence for DFS or death for OS occurred within 5 years, and as no event by 5 years if the patient remained event-free at 5 years, including when an event occurred after 5 years. Patients with no event but follow-up shorter than 5 years were treated as right-censored and excluded from the 5-year binary end point analyses to avoid outcome misclassification. For time-to-event analyses of DFS, events were defined as recurrence or death, whichever occurred first, and patients without an event were censored at the last date known to be alive and disease-free. For time-to-event analyses of OS, the event was death from any cause, and patients without death were censored at the last known alive date.

In step 2, we also adopted an end-to-end deep learning approach in which the model directly analyzes the entire CT image without prior segmentation of the tumor on the CT. The 3D ResNet-18 architecture was used as the backbone, as described in step 1. Additionally, a supervised machine-learning model was developed using clinical data as input to estimate same outcomes. The outputs of both models were integrated to calculate final prediction. Patient-wise independence between model development and evaluation was ensured using the out-of-bag bootstrap framework, where each iteration defines an in-bag bootstrap sample and a mutually exclusive out-of-bag evaluation sample.

Model performance was evaluated using bootstrap resampling with 1000 iterations. In each iteration, the model was fitted using the bootstrap sample and evaluated on the corresponding out-of-bag sample, thereby separating model fitting from performance evaluation. The performance metrics included sensitivity, specificity, positive predictive-value, negative-predictive value, and ROC-AUC. ROC-AUC was used to summarize discrimination. For threshold-dependent metrics, the decision threshold was determined by maximizing the Youden index using only the bootstrap sample within each iteration and was then applied unchanged to the corresponding out-of-bag sample for evaluation, thereby minimizing optimism bias due to threshold tuning. We report 95% CI for all principal performance metrics using 1000 bootstrap iterations; AUC CIs were obtained using standard bootstrap resampling, whereas sensitivity, specificity, positive predictive-value, and negative-predictive value were obtained using the out-of-bag bootstrap procedure described above.

### Ethical Considerations

This study was conducted in accordance with the ethical principles outlined in the Declaration of Helsinki and was approved by the Institutional Review Board of the National Cancer Center Hospital East in Japan (approval No. 2022-165, dated September 2022). Written informed consent for publication was obtained from patients in Cohort 2. An opt-out applied to patients in cohort 1 and 2 who had difficulty providing consent.

## Results

### Patient Demographics

**Figure 2:**
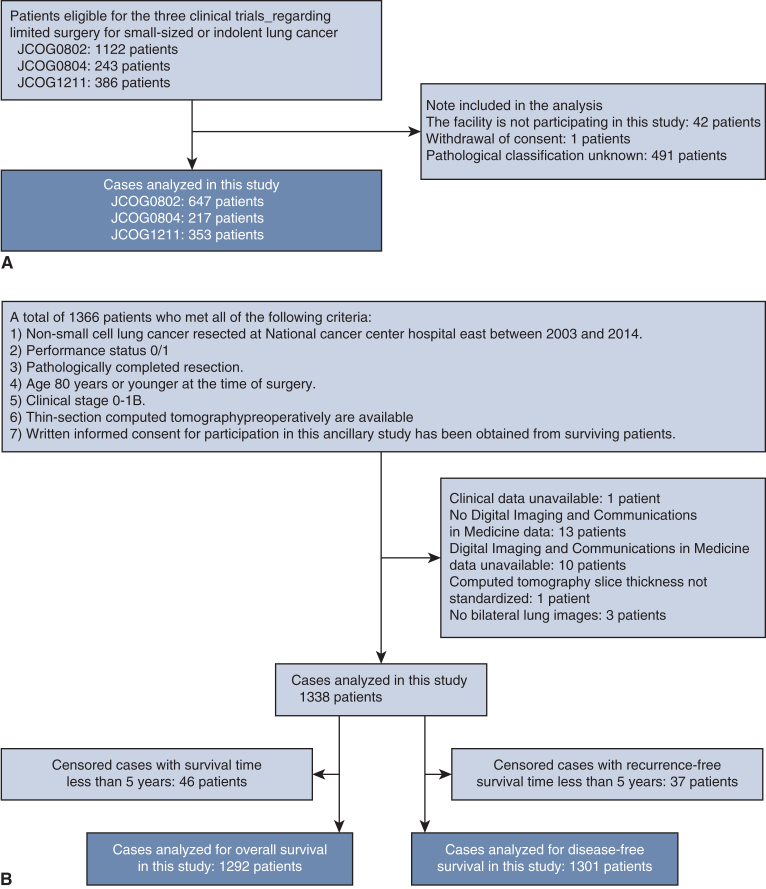
Patient flow diagram for cohort 1 in step 1 (A) and cohort 2 in step 2 (B). *JCOG*, Japan Clinical Oncology Group.

In step 2, 1338 patients were included in the final analytic dataset, and 1301 and 1292 patients were eligible for the 5-year binary DFS and OS analyses, respectively, after excluding patients without events and with follow-up shorter than 5 years. Figure 2 shows the patient-flow diagram, and Table 1 presents the background information of the participants analyzed in cohort 1. Of the 1217 patients included in this study, 514 (42.2%) were men, and the median age (interquartile range [IQR] Q1-Q3) at enrollment was 66 years (IQR, 60-71 years). Histological classifications according to the fourth edition of the WHO classification were available for 353 study participants (29.0%). These participants had minimally invasive adenocarcinoma (n = 119 [33.7%]), adenocarcinoma in situ (n = 89 [25.2%]), predominantly lepidic adenocarcinoma (n = 85 [24.1%]), predominantly papillary adenocarcinoma (n = 44 [12.5%]), predominantly acinar adenocarcinoma (n = 10 [2.8%]), atypical adenomatous hyperplasia (n = 2 [0.6%]), predominantly micropapillary adenocarcinoma (n = 2 [0.6%]), predominantly solid adenocarcinoma (n = 1 [0.3%]), or invasive mucinous adenocarcinoma (n = 1 [0.3%]). Histological classifications according to the Noguchi classification were available for 864 patients (71.0%). Among these 864 patients, Type C was most common (n = 428 [49.5%]), followed by B (n = 155 [17.9%]), F (n = 127 [14.7%]), A (n = 83 [9.6%]), E (n = 45 [5.2%]), and D (n = 26 [3.0%]).

**Table 1:** Patients’ clinical background

Patient characteristics	Step 1 cohort (n = 1217)	Step 2 cohort (5-y DFS, n = 1301)	Step 2 cohort (5-y OS, n = 1292)	*P* value for comparison of STEP1 and STEP2 RFS cohorts	Test method
Sex					
N	1217	1301	1292	<.001	χ^2^ test
Male	514 (42.2)	720 (55.3)	716 (55.4)		
Female	703 (57.8)	581 (44.7)	576 (44.6)		
Unknown	0 (0.0)	0 (0.0)	0 (0.0)		
Age (y)					
N	1217	1301	1292	.016	Welch *t* test
Mean ± SD	64.72 ± 9.32	65.58 ± 8.49	65.58 ± 8.49		
Median	66	67	67		
Q1, Q3	60.00, 71.00	60.00, 72.00	60.00, 72.00		
Min, Max	24.00, 85.00	33.00, 79.00	33.00, 79.00		
Smoking history					
N	1217	1301	1292	<.001	χ^2^ test
Never smoker	669 (55.0)	526 (40.4)	522 (40.4)		
Current or Smoker	548 (45.0)	775 (59.6)	770 (59.6)		
Unknown	0 (0.0)	0 (0.0)	0 (0.0)		
Smoking index					
Average no. of cigarettes per day					
N	542	1301	1292	<.001	Welch *t* test
Mean ± SD	22.09 ± 14.20	14.07 ± 16.41	14.07 ± 16.43		
Median	20	12	11		
Q1, Q3	15.00, 30.00	0.00, 20.00	0.00, 20.00		
Min, Max	1.00, 200.00	0.00, 200.00	0.00, 200.00		
Years of smoking					
N	543	1301	1292	<.001	Welch *t* test
Mean ± SD	31.06 ± 14.27	21.64 ± 20.80	21.63 ± 20.80		
Median	33	20	20		
Q1, Q3	20.00, 42.00	0.00, 41.00	0.00, 41.00		
Min, Max	1.00, 60.00	0.00, 62.00	0.00, 62.00		
Pack-years					
N	540	1301	1292	<.001	Welch *t* test
Mean ± SD	36.24 ± 27.28	26.39 ± 32.30	26.36 ± 32.31		
Median	32	15	15		
Q1, Q3	16.00, 49.00	0.00, 45.00	0.00, 45.00		
Min, Max	0.05, 208.00	0.00, 232.00	0.00, 232.00		
Brinkman index					
N	543	1301	1292	<.001	Welch *t* test
Mean ± SD	774.95 ± 865.71	527.71 ± 646.06	527.23 ± 646.27		
Median	640	300	300		
Q1, Q3	320.00, 990.00	0.00, 900.00	0.00, 900.00		
Min, Max	1.00, 9801.00	0.00, 4640.00	0.00, 4640.00		
Tumor location					
N	1217	1301	1292	<.001	χ^2^ test
Right upper lobe	373 (30.6)	448 (34.4)	446 (34.5)		
Right middle lobe	6 (0.5)	102 (7.8)	101 (7.8)		
Right lower lobe	295 (24.2)	268 (20.6)	268 (20.7)		
Left upper lobe	362 (29.7)	305 (23.4)	301 (23.3)		
Left lower lobe	181 (14.9)	178 (13.7)	176 (13.6)		
ECOG PS					
N	1217	1301	1292	<.001	Fisher exact test
0	1192 (97.9)	1188 (91.3)	1182 (91.5)		
1	25 (2.1)	110 (8.5)	107 (8.3)		
2	0 (0.0)	3 (0.2)	3 (0.2)		
3	0 (0.0)	0 (0.0)	0 (0.0)		
4	0 (0.0)	0 (0.0)	0 (0.0)		
Preoperative FEV1					
N	1217	1300	1291	.728	Welch *t* test
Mean ± SD	2338.82 ± 582.90	2325.34 ± 1260.63	2327.13 ± 1263.86		
Median	2260	2220	2220		
Q1, Q3	1930.00, 2700.00	1830.00, 2670.00	1830.00, 2670.00		
Min, Max	1130.00, 4490.00	660.00, 32,000.00	660.00, 32,000.00		
Preoperative FVC					
N	1217	1300	1291	<.001	Welch *t* test
Mean ± SD	3072.98 ± 755.75	2965.19 ± 746.17	2967.38 ± 745.20		
Median	2980	2890	2890		
Q1, Q3	2520.00, 3550.00	2437.50, 3452.50	2440.00, 3455.00		
Min, Max	1370.00, 6050.00	1270.00, 6800.00	1270.00, 6800.00		
Preoperative serum CEA					
N	1208	1301	1292	.092	Welch *t* test
Mean ± SD	2.72 ± 2.51	7.68 ± 106.09	7.67 ± 106.46		
Median	2.1	2.9	2.9		
Q1, Q3	1.40, 3.10	1.80, 5.00	1.80, 5.00		
Min, Max	0.10, 30.40	0.00, 3820.00	0.00, 3820.00		
Preoperative CT findings					
Preoperative maximum tumor diameter, including ground glass component (cm)					
N	1217	1301	1292	<.001	Welch's t-test
Mean ± SD	1.60 ± 0.46	2.51 ± 0.94	2.50 ± 0.94		
Median	1.6	2.4	2.4		
Q1, Q3	1.30, 1.90	1.80, 3.10	1.80, 3.10		
Min, Max	0.59, 3.00	0.70, 18.00	0.70, 18.00		
Maximum diameter of preoperative consolidation					
N	1217	1301	1292	<.001	Welch *t* test
Mean ± SD	0.82 ± 0.57	2.08 ± 0.96	2.08 ± 0.96		
Median	0.8	2	2		
Q1, Q3	0.36, 1.20	1.40, 2.80	1.40, 2.80		
Min, Max	0.00, 2.00	0.00, 4.40	0.00, 4.40		
Pleural indentation					
N	1217	N.A.	N.A.	N.A.	N.A.
Absent	761 (62.5)	N.A.	N.A.		
Present	456 (37.5)	N.A.	N.A.		
Air bronchogram					
N	353	N.A.	N.A.	N.A.	N.A.
Absent	205 (58.1)	N.A.	N.A.		
Present	148 (41.9)	N.A.	N.A.		
Consolidation to tumor ratio					
N	1217	1301	1292	<.001	Welch *t* test
Mean ± SD	0.52 ± 0.35	0.83 ± 0.27	0.83 ± 0.27		
Median	0.47	1	1		
Q1, Q3	0.23, 0.89	0.70, 1.00	0.69, 1.00		
Min, Max	0.00, 1.00	0.00, 1.00	0.00, 1.00		
WHO classification of the tumors of the lung					
N	953	1301	1292	<.001	Fisher exact test
Non–small cell carcinoma: Unspecified	0 (0.0)	0 (0.0)	0 (0.0)		
Preinvasive lesion	0 (0.0)	0 (0.0)	0 (0.0)		
Squamous cell carcinoma	0 (0.0)	150 (11.5)	148 (11.5)		
Small cell carcinoma	0 (0.0)	0 (0.0)	0 (0.0)		
Combined small cell carcinoma	0 (0.0)	0 (0.0)	0 (0.0)		
Adenocarcinoma	953 (100.0)	1081 (83.1)	1076 (83.3)		
Large cell carcinoma	0 (0.0)	21 (1.6)	21 (1.6)		
Large cell neuroendocrine carcinoma	0 (0.0)	19 (1.5)	18 (1.4)		
Adenosquamous carcinoma	0 (0.0)	16 (1.2)	15 (1.2)		
Pleomorphic, sarcomatoid, or carcinoma containing sarcomatous components	0 (0.0)	4 (0.3)	4 (0.3)		
Carcinoid tumors	0 (0.0)	0 (0.0)	0 (0.0)		
Salivary gland-type tumors	0 (0.0)	0 (0.0)	0 (0.0)		
Unclassified carcinoma	0 (0.0)	0 (0.0)	0 (0.0)		
Other carcinoma	0 (0.0)	10 (0.8)	10 (0.8)		
Noncancerous lesion	0 (0.0)	0 (0.0)	0 (0.0)		
Histological subtype of adenocarcinoma					
N	353	1301	1301	<.001	χ^2^ test
Atypical adenomatous hyperplasia	2 (0.6)	0 (0.0)	0 (0.0)		
Adenocarcinoma in situ	89 (25.2)	88 (6.8)	88 (6.8)		
Minimally invasive adenocarcinoma	119 (33.7)	0 (0.0)	0 (0.0)		
Lepidic adenocarcinoma	85 (24.1)	362 (27.8)	362 (28.0)		
Acinar adenocarcinoma	10 (2.8)	154 (11.8)	152 (11.8)		
Papillary adenocarcinoma	44 (12.5)	305 (23.4)	302 (23.4)		
Micropapillary adenocarcinoma	2 (0.6)	12 (0.9)	12 (0.9)		
Solid adenocarcinoma	1 (0.3)	159 (12.2)	159 (12.3)		
Invasive mucinous adenocarcinoma	1 (0.3)	0 (0.0)	0 (0.0)		
Colloid adenocarcinoma	0 (0.0)	1 (0.1)	1 (0.1)		
Fetal adenocarcinoma	0 (0.0)	0 (0.0)	0 (0.0)		
Enteric adenocarcinoma	0 (0.0)	1 (0.1)	1 (0.1)		
Tumor differentiation					
N	864	1301	1292	<.001	Fisher exact test
Well differentiation	538 (62.3)	393 (30.2)	393 (30.4)		
Moderate differentiation	199 (23.0)	676 (52.0)	670 (51.9)		
Poor differentiation	40 (4.6)	156 (12.0)	155 (12.0)		
Unknown	87 (10.1)	76 (5.8)	74 (5.7)		
Noguichi's classification					
N	864	N.A.	N.A.	N.A.	N.A.
Type A	83 (9.6)	N.A.	N.A.		
Type B	155 (17.9)	N.A.	N.A.		
Type C	428 (49.5)	N.A.	N.A.		
Type D	26 (3.0)	N.A.	N.A.		
Type E	45 (5.2)	N.A.	N.A.		
Type F	127 (14.7)	N.A.	N.A.		
Unknown	0 (0.0)	N.A.	N.A.		
EGFR mutation					
N	N.A.	1301	1292	N.A.	N.A.
Absent	N.A.	322 (24.8)	319 (24.7)		
Present	N.A.	288 (22.1)	286 (22.1)		
Unknown	N.A.	691 (53.1)	687 (53.2)		

Values are presented as n (%) unless otherwise noted. *DFS*, Disease-free survival; *OS*, overall survival; *RFS*, recurrence-free survival; *ECOG*, Eastern Cooperative Oncology Group Performance Status; *FEV1*, forced expiratory volume in 1 second; *CEA*, carcinoembryonic antigen; *CT*, computed tomography; *N.A.*, not available; *WHO*, World Health Organization; *EGFR*, epidermal growth factor receptor.

The background information of the participants analyzed in cohort 2 is shown in Table 1. When analyzing 5-year DFS and 5-year OS rates as different groups with data available for each, we noted 720 (55.3%) and 716 (55.4%) men, with both groups having a median age at registration of 67 years (IQR, 60-72 years). The numbers of patients with epidermal growth factor receptor mutations in each group were 288 (22.1%) and 286 (22.1%), respectively.

### Pathological Classification Predictions

The performance evaluation indices of the constructed models (1-13) are shown in Online Data Supplement, and ROC curves of each model are shown in Online Data Supplement (models 1-3), *B* (models 4–6), *C* (models 7–9), *D* (model 11), *E* (model 12), and *F* (model 13). The confusion matrix for the classification of each tissue in the evaluation data is shown in Online Data Supplement (model 10). In addition, considering the pathological findings, each pathological classification was used as the output of each model according to the WHO and Noguchi classifications (Online Data Supplement). When the output was whether or not the tumor was classified according to the WHO classification of minimally invasive adenocarcinoma and more malignant subtypes or the Noguchi classification of type B and more malignant subtypes (output 1 in Online Data Supplement), the AUC was 0.784 for the late-fusion model (model 3) and 0.520 for the early-fusion model (model 11). When the output was whether or not the tumor was classified according to the WHO classification of predominantly lepidic adenocarcinoma and more malignant subtypes or the Noguchi classification of type C and more malignant subtypes (output 2 in Online Data Supplement), the AUC was 0.787 for the late-fusion model (model 6) and 0.501 for the early-fusion model (model 12). When the output (output 3 in Online Data Supplement) was the WHO classification of predominantly acinar adenocarcinoma and more malignant subtypes or the Noguchi classification of type D and more malignant subtypes, the AUC was 0.745 for the late-fusion model (model 9) and 0.510 for the early-fusion model (model 13). With the late-fusion model, models 3, 6, and 9 were used as inputs, and the WHO classification or Noguchi classification was used as the output; a quadratic Kappa value of 0.492 was obtained from the confusion matrix for each histological classification (Online Data Supplement).

### Prognosis and Recurrence Prediction

#### Five-year DFS prediction model DFS curves according to risk group

**Figure 3:**
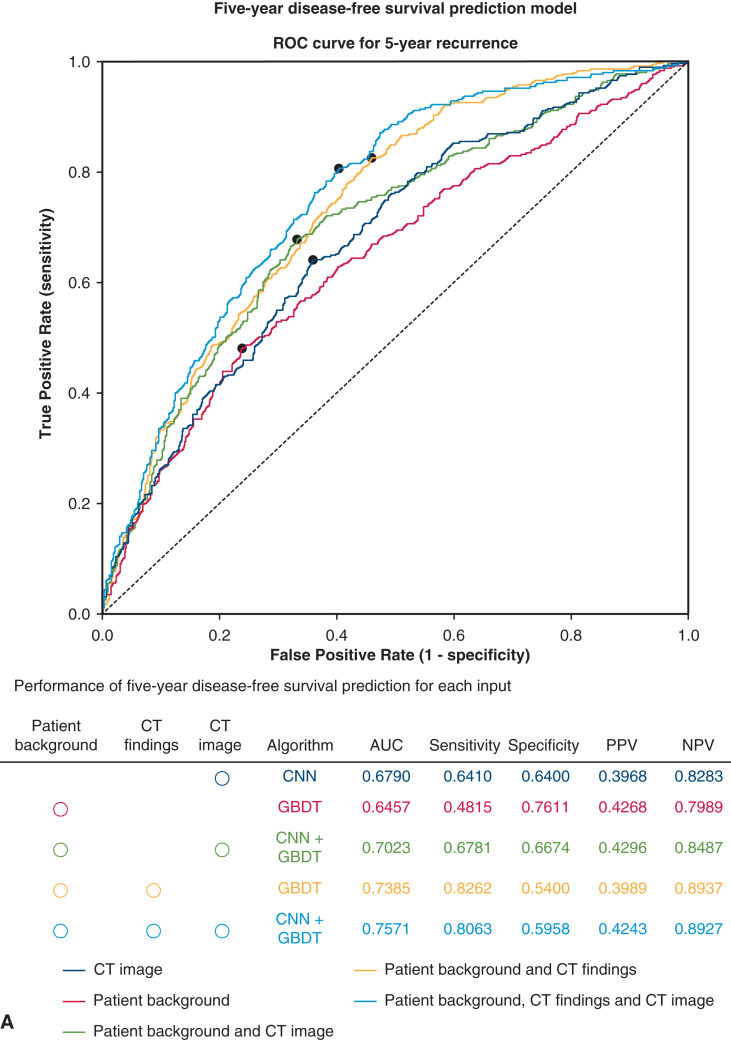

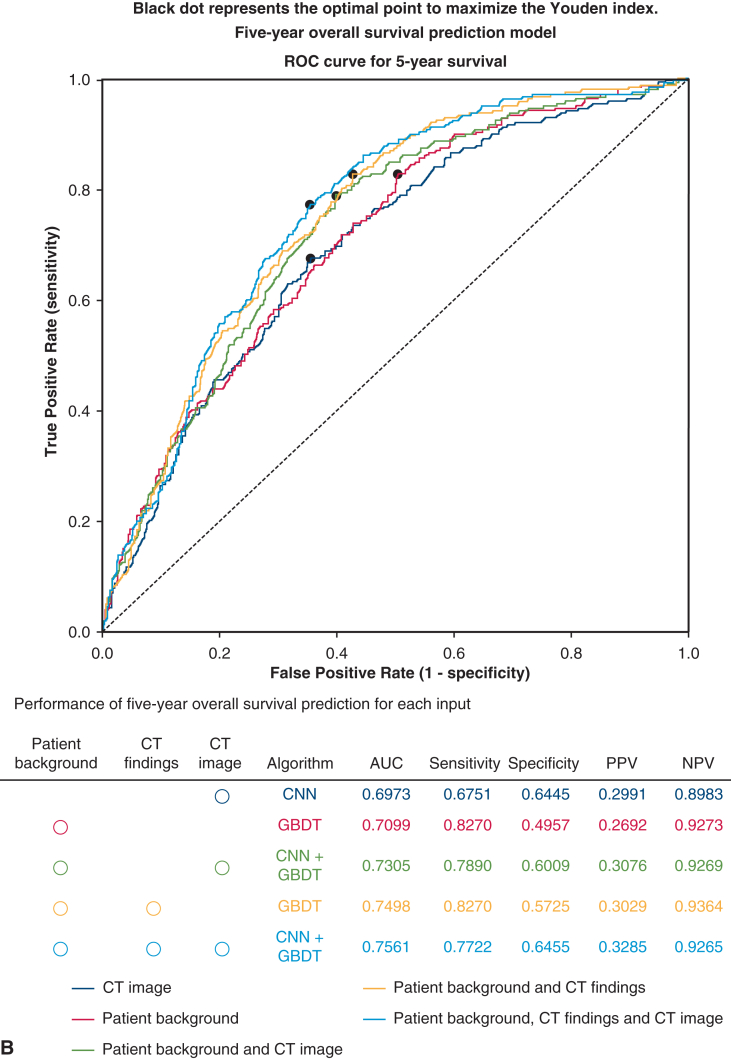
Comparison of predictive model performance. Performance of the 5-year disease-free survival (*DFS*) prediction model (A) and 5-year overall survival (*OS*) prediction model (B), with each explanatory factor. The computed tomography (*CT*) findings reflect a physician's assessment of thin-section CT images. The CT image reflects the output of a machine-learning model developed using CT images as input data. Patient background refers to clinical factors such as sex and smoking habits and values obtained from testing, excluding the CT findings and images. *AUC*, Area under the curve; *PPV*, positive predictive value; *NPV*, negative predictive value.

We established 5-year DFS-prediction models by analyzing different combinations of explanatory factors. The predictive performance of each model and the associated ROC curves are shown in Figure 3, *A*. The AUC of the 5-year DFS prediction models ranged from 0.6457 to 0.7571. A model based on a combination of patient background, CT findings by the attending physician, and AI-based images showed the best predictive performance, with an AUC of 0.7571, with 95% CI for all principal metrics reported in Online Data Supplement.

**Figure 4:**
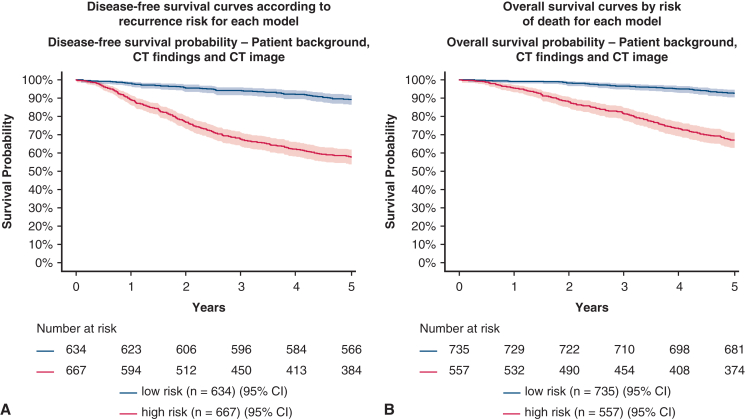
Risk-specific survival curves. A, Disease-free survival (*DF*) curves predicted by each model. B, Overall survival (*OS*) curves for each model. *CT*, Computed tomography.

For each 5-year DFS-prediction model created, the predicted value that maximized the Youden index was used as the threshold, and patients were divided into 2 groups—a high-risk group and a low-to-intermediate-risk group. Threshold-dependent metrics were evaluated using the out-of-bag bootstrap procedure described in the Methods section, and the corresponding 95% CIs are reported in Online Data Supplement. Survival curves were plotted with recurrence or death as events (Online Data Supplement). The respective 5-year DFS rates in the high-risk and low-to-intermediate-risk groups, as predicted by each model, were as follows: 0.603 and 0.828 (CT image model), 0.573 and 0.799 (patient background model), 0.570 and 0.849 (patient background + CT image model), 0.601 and 0.894 (patient background + CT findings), and 0.576 and 0.893 (patient background + CT findings + CT image model), as shown in Figure 4, *A*.

#### Five-year OS prediction model and OS rates, stratified by risk group

We created 5-year OS-prediction models by testing various combinations of explanatory factors. The predictive performance of each model is shown in Figure 3, *B*, and the corresponding ROC curves are shown in Figure 3, *B*. The AUC values of the 5-year OS-prediction models ranged from 0.6973 to 0.7561. A combined model based on the patient background, CT findings by the attending physician, and AI-based images showed the best predictive performance, with an AUC of 0.7561, with 95% CI for all principal metrics reported in Online Data Supplement.

For each 5-year OS-prediction model, the predicted value that maximized the Youden index was set as the threshold, and survival curves were plotted with death as an event that was used to divide the patients into 2 groups, as described above for the DFS-prediction models (Figure 3, B). Threshold-dependent metrics were evaluated using the out-of-bag bootstrap procedure described in the Methods section, and the corresponding 95% CIs are reported in Online Data Supplement. The respective 5-year OS rates in the high-risk and low-to-intermediate-risk groups, as predicted by each model, were as follows: 0.701 and 0.898 (CT image model), 0.731 and 0.927 (patient background model), 0.692 and 0.927 (patient background + CT image model), 0.697 and 0.936 (patient background + CT findings), and 0.671 and 0.927 (patient background + CT findings + CT image model), as shown in Figure 4, *B*.

#### Influence of each factor on the outcome

The contribution of each variable to the predictive performance of 5-year DFS is shown in Online Data Supplement. Little improvement in predictive accuracy was observed as a function of the surgical procedure when the patient background and CT findings were included. The presence or absence of epidermal growth factor receptor mutations had a little effect on the predictive accuracy when patient background, CT findings, and pathological findings were included.

When the CT image model was not included, removing the histological subtype and Noguchi classification factors reduced the AUC (Online Data Supplement). When the CT image model was included, removing the histological subtype reduced the AUC, although removing the Noguchi classification did not (Online Data Supplement).

## Discussion

By developing an algorithm that predicts pathological findings in advance, patient outcomes could be predicted more accurately. In step 1, across all tasks, the late-fusion model achieved a higher AUC than the early-fusion model both when using clinical data alone and when using CT images alone. Moreover, combining the CT image model with clinical-data model yielded higher AUCs than either model alone, suggesting an added value of multimodal integration. There remains room to further improve the CT-only model. Various strategies have been explored to enhance the predictive performance of CT-based deep learning. Kinetics-600, a large-scale video dataset commonly used in machine learning and deep learning for action recognition, is typically used to train convolutional neural networks and transformer-based models.^24^, ^25^, ^26^ However, in step 2, pretraining with Kinetics-600 did not improve performance compared with the step 1 model and was therefore not adopted (data not shown). We also developed a model that extracted the tumor area as an ROI in preoperative CT scans, but it was inferior to a model that analyzed the entire image (data not shown). Because ROI definition is subjective and tumor boundaries are often difficult to delineate. In addition, prognostically relevant information may exist outside the tumor region. Therefore, we adopted an end-to-end deep learning framework that analyzes the entire preoperative CT volume without requiring a priori tumor segmentation. Several prior CT-based prognostic deep learning studies have relied on manual lesion localization or user-defined tumor ROIs to construct model inputs, which can be labor-intensive and may limit scalability and reproducibility across readers and institutions.^27^, ^28^, ^29^ This setting is inherently more demanding than conventional radiomics pipelines, which typically rely on manual or semiautomatic delineation of the tumor region and thus incorporate explicit lesion localization. Despite the absence of tumor contours, our model achieved discrimination comparable to that reported in prior radiomics-based studies for resected stage I NSCLC, where AUCs of approximately 0.70 to 0.75 have been described for predicting postoperative recurrence or survival.^30^^,^^31^ These findings suggest that an end-to-end approach may capture prognostically relevant imaging signatures from CT beyond explicitly segmented tumor regions, potentially including peritumoral and whole lung features. From a clinical perspective, eliminating the segmentation step could improve scalability and reduce interobserver variability, supporting the feasibility of the proposed approach as a complementary tool for postoperative risk stratification.

Although clinical decision making routinely incorporates both imaging and clinical information, we included a CT-only model for 2 reasons. First, methodologically, we evaluated models with and without CT images to quantify how much prognostic information CT contributes and to show how prediction performance changes when CT and clinical data are used together compared with either modality alone. Second, practically, it provides a segmentation-free baseline that remains usable in real-world settings, such as when clinical variables are partially missing, recorded in different formats across institutions, or only available later in the workflow rather than at the time of imaging. Moreover, evaluating a CT-only model enables assessment of whether CT images encode prognostic information beyond physician-interpreted CT findings. Accordingly, the CT-only results are not presented as a standalone clinical workflow, but rather as a benchmark for comparison.

As shown in Figure 3, the predictive performance of the CT imaging model alone achieved an AUC of 0.6790 for 5-year DFS predictions and 0.6973 for 5-year OS predictions. To develop a more accurate predictive model, we integrated 2 additional explanatory factors: preoperative information and CT findings assessed by a physician. This combination improved the predictive performance, yielding an AUC of 0.7571 for 5-year DFS predictions and an AUC of 0.7561 for 5-year OS predictions.

Notably, there is no universally accepted threshold defining the clinical meaningfulness of small AUC differences; nevertheless, AUC is a standard summary measure of discrimination and is useful for quantifying incremental changes in performance as predictors are added. Importantly, the proposed model is not intended to replace clinicians’ judgment or established prognostic factors, but rather to complement preoperative risk stratification. Therefore, the improvement achieved by integrating CT images with clinical information (eg, AUC 0.679-0.697 for CT-only vs ∼0.756 for the integrated model) should be interpreted in terms of how it could support clinical actions, such as selecting patients for closer surveillance or considering preoperative therapy. In addition, because AUC summarizes performance across all possible thresholds, it should be complemented by evaluations at clinically relevant decision thresholds and by assessing risk-group separation, as shown in the Kaplan-Meier analyses.

The pretrained CT imaging model complemented physician-assessed CT-based T-factor evaluation for prognostic estimation. This finding indicates that, although the predictive performance of the constructed CT imaging model was not sufficient to completely replace existing prognostic parameters, it complemented and reinforced the prediction accuracy.

Furthermore, for 5-year DFS predictions, combining models based on preoperative information, CT findings by a physician, and CT imaging achieved an AUC of 0.7571, whereas replacing the CT imaging model with the pathological findings model yielded an AUC of 0.7686. These results suggested that the CT imaging model provided prognostic performance comparable to pathological findings, which may be relevant for informing future preoperative treatment strategies.

A threshold was set to maximize the Youden index in the model that included all 3 factors, and survival was compared by classifying the patients into 2 groups. The 5-year DFS rates were 57.6% and 89.3% in the high- and low-to-intermediate-risk groups, respectively, and the 5-year OS rates were 67.1% and 92.7%, respectively. These results indicate that a prediction model based on combining the predicted values from a CT image model with existing preoperative factors can effectively stratify clinical stage I patients based on their prognosis, suggesting that patients in the high-risk group may be suitable targets for future treatments as they are developed.

This study had some limitations. The cohort consisted solely of Japanese patients, and the clinical trial data used in step 1 were evaluated using the outdated Noguchi classification scheme, which had to be converted to the WHO classification scheme. Additionally, the real-world data used in step 2 was obtained from a single institution. In addition, external validation in independent, multi-institutional cohorts is needed to confirm generalizability. Prospective studies will be required to determine whether model-guided decision making changes management and improve outcomes.

Overall, incorporating an AI-based, end-to-end CT imaging model that analyzes the entire preoperative CT volume without tumor segmentation alongside established preoperative factors enabled more accurate prognostic estimation. This end-to-end imaging assessment may facilitate preoperative identification of high-risk patients and provides a scalable framework that can support selection of candidates for preoperative treatment and refinement of therapeutic strategies. A practical near-term use case is risk enrichment and stratification in prospective studies; for example, the model could identify patients at high predicted risk and support enrollment or stratification in neoadjuvant therapy trials after resection. Such an approach may improve trial efficiency by enriching for events while maintaining standard-of-care management.

### Data Sharing

The data generated in this study are not publicly available due to contractual and informed-consent restrictions, but they can be accessed from the corresponding author upon reasonable request.

## Conflict of Interest Statement

Dr Aokage is supported by the ESR program from AstraZeneca (grant No. ESR-20-21080) and has received grant funding paid to his institution from the National Cancer Center (National Cancer Center Research and Development Fund grant Nos. 2020-J-3 and 2023-J-03), as well as from the Japan Agency for Medical Research and Development under the Project for the Practical Application of Innovative Cancer Medical Care: Research on the Establishment of Standard Function-Preserving Surgery for Non-Invasive or Small-Sized Non–Small Cell Lung Cancer. Dr Aokage has also received consulting fees from AstraZeneca and Chugai Pharmaceutical Co Ltd. He has received honoraria for lectures from AstraZeneca KK, Bristol-Myers Squibb, Care-net, Chugai Pharmaceutical Co Ltd, Covidien Japan, CSL Behring, Daiichi Sankyo, Inc, Eli Lilly Japan KK, Guardant Health, Japan Blood Products Organization, Johnson & Johnson KK, Kyowa Kirin, Olympus Corporation, Ono Pharmaceutical Co Ltd, and Phase One and has received honoraria for lectures and/or manuscript writing from TAIHO Pharmaceutical Co Ltd, Teijin Healthcare, Medical Tribune KK, MSD, Kanehara Publisher, and Medical View Corporation. Dr Tsuboi is supported by funding from AstraZeneca KK for the present work, has received commissioned research grant funding paid to his institution from MSD, AstraZeneca KK, Ono Pharmaceutical Co Ltd, Bristol-Myers Squibb KK, Eli Lilly Japan, MiRXES Japan, and Johnson & Johnson Japan; has received honoraria for lectures from Johnson & Johnson Japan, AstraZeneca KK, Chugai Pharmaceutical Co Ltd, Taiho Pharma, Medtronic Japan, Ono Pharmaceutical Co Ltd, MSD, Bristol-Myers Squibb KK, Daiichi-Sankyo, and Amgen KK; serves in advisory roles for AstraZeneca KK and MSD, and as a member of the Data Safety Monitoring Board for Chugai Pharmaceutical Co Ltd; and holds a leadership position as a board director of the Japan Lung Cancer Society. Dr Ukita has received consulting fees from Emeraid Inc and Fvital Inc. Dr Ogaki is employed by M3 Inc and holds stock options in the company and serves as a director at M3 AI Inc. Dr Miura is employed by M3 Inc and Matsuo Institute Inc, and holds stock options in M3 Inc. All other authors reported no conflicts of interest.

The *Journal* policy requires editors and reviewers to disclose conflicts of interest and to decline handling or reviewing manuscripts for which they may have a conflict of interest. The editors and reviewers of this article have no conflicts of interest.
