# Supplementary material for: Artificial intelligence-based algorithm for predicting outcomes in early-stage lung cancer: An annotation-free imaging artificial intelligence study
Source: JTCVS Open. 2026 Mar 30;31:101742. doi: 10.1016/j.xjon.2026.101742 (PMC13316326; doi:10.1016/j.xjon.2026.101742)
Supplement: Online Data Supplement [file mmc1.pptx]

## Slide 1
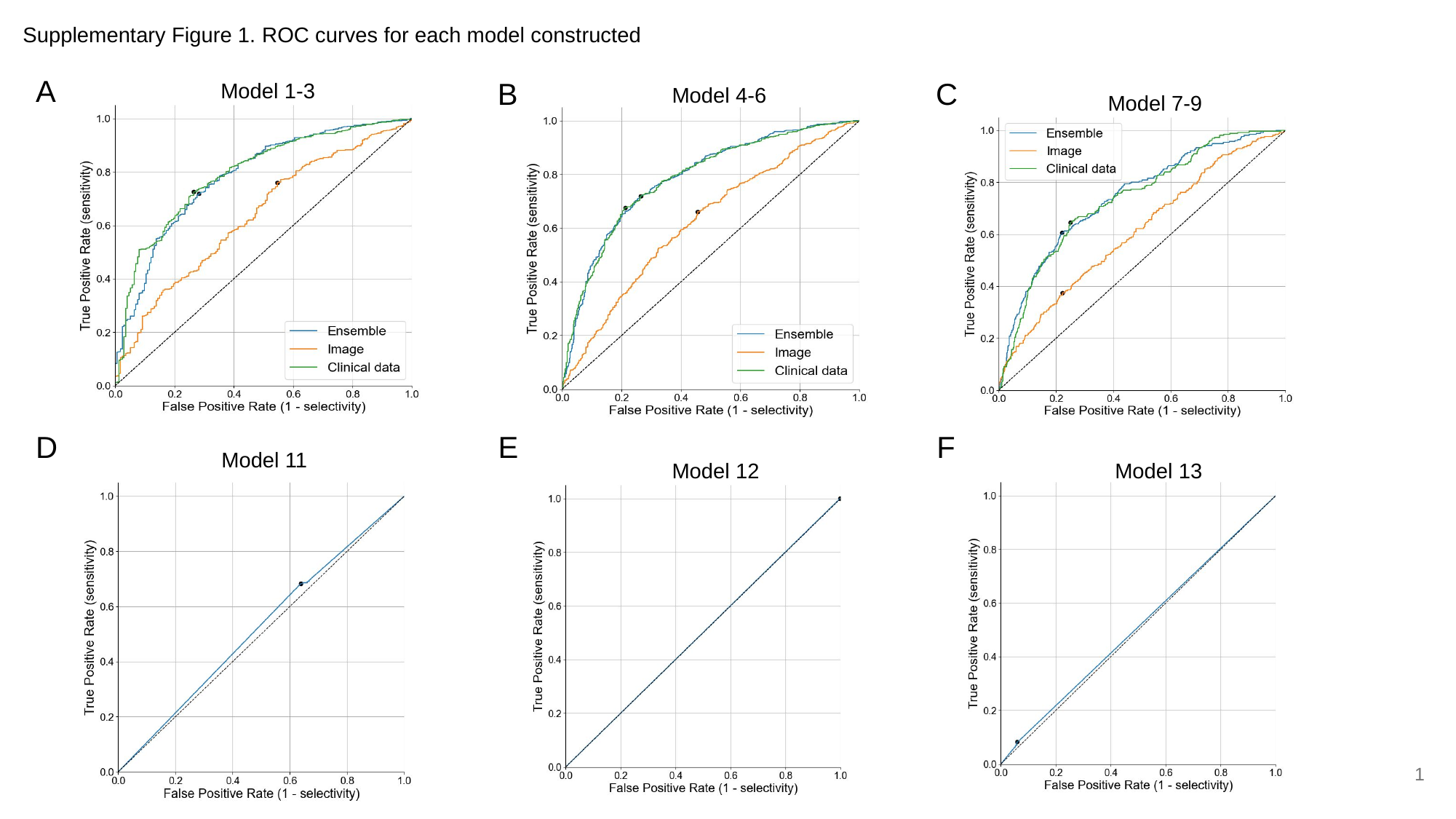

Supplementary Figure 1. ROC curves for each model constructed
A
B
C
Model 1-3
Model 4-6
Model 7-9
D
E
F
Model 11
Model 12
Model 13
1

## Slide 2
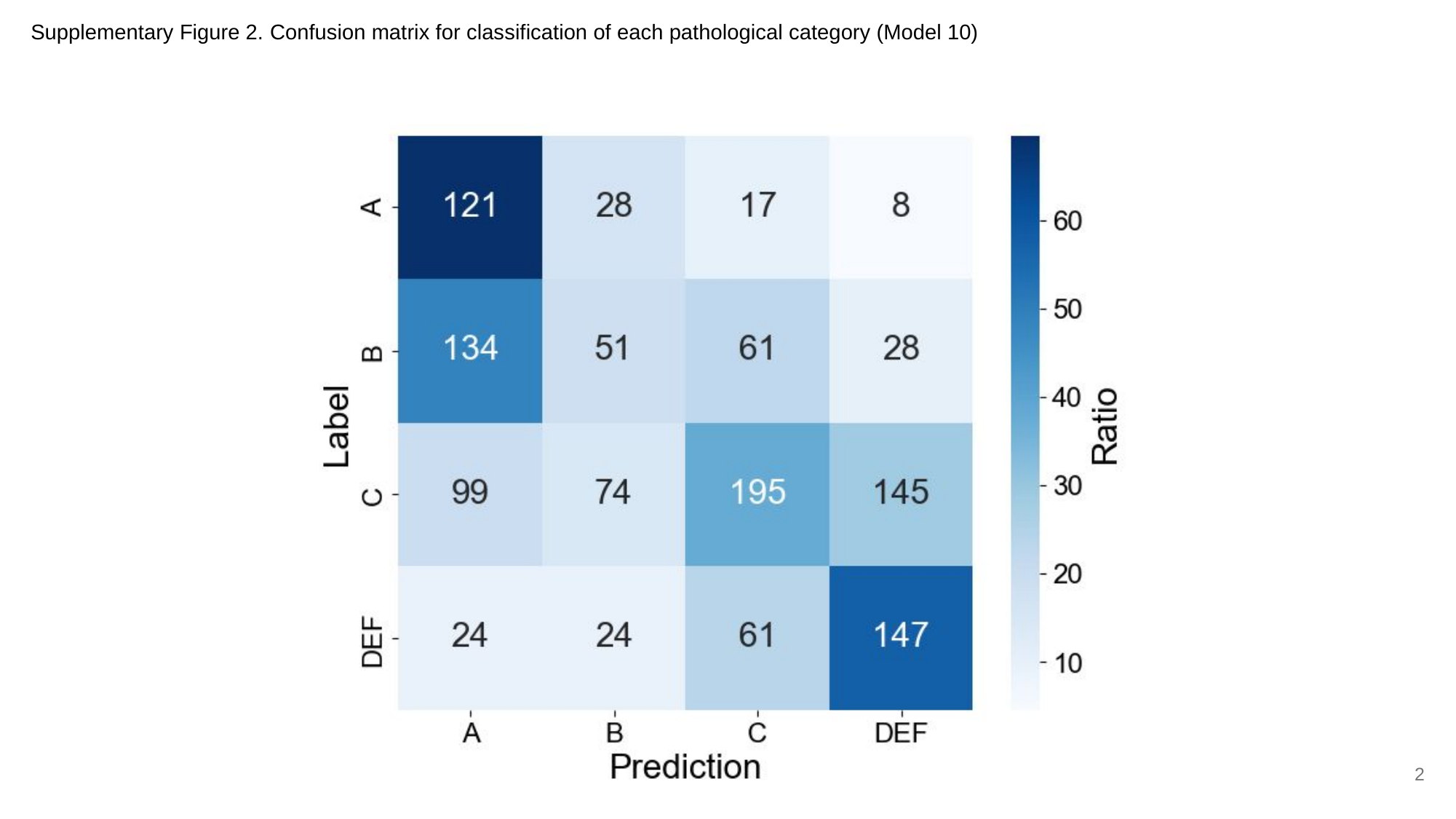

Supplementary Figure 2. Confusion matrix for classification of each pathological category (Model 10)
2

## Slide 3
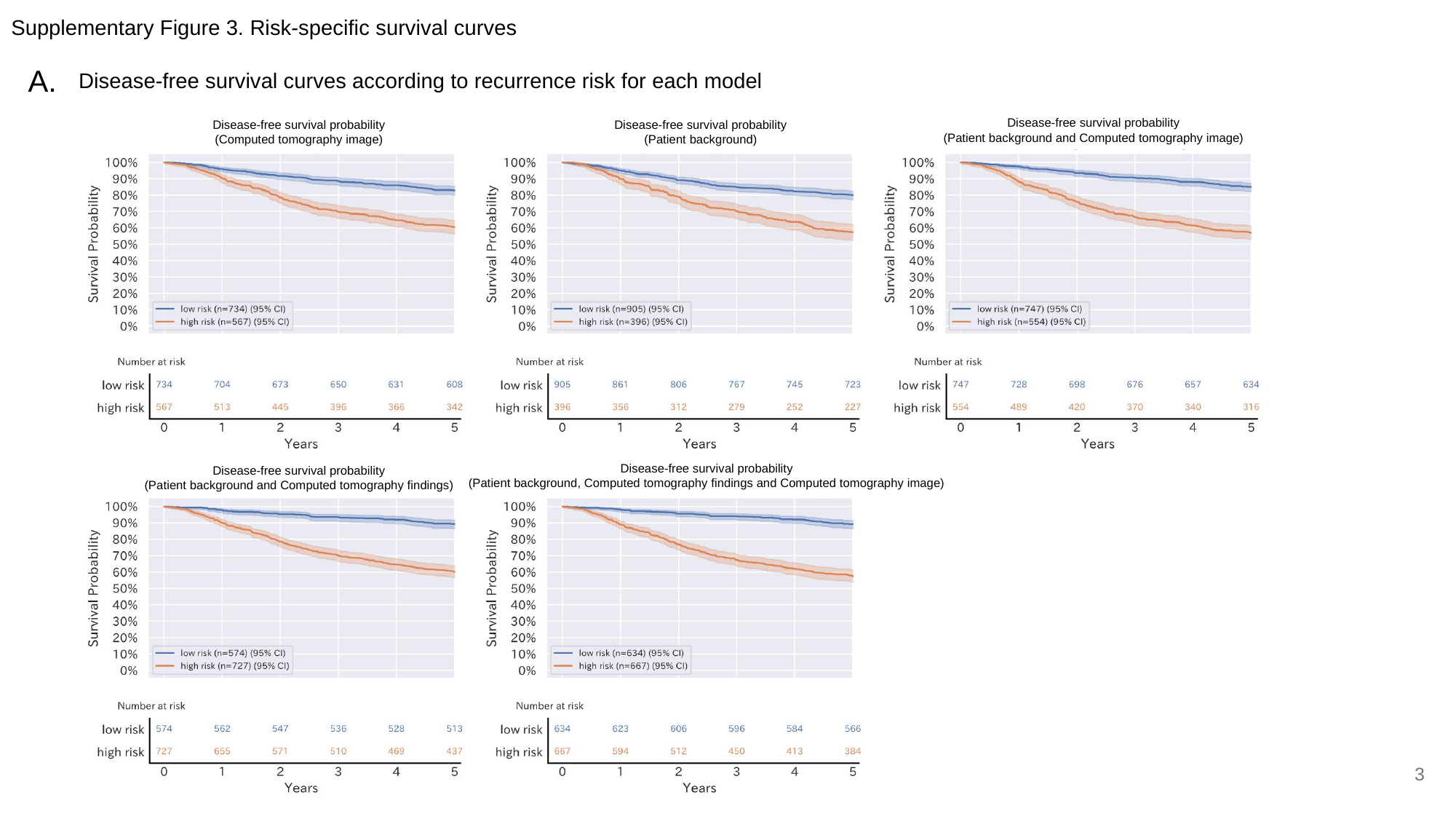

Supplementary Figure 3. Risk-specific survival curves
A.
Disease-free survival curves according to recurrence risk for each model
Disease-free survival probability
(Patient background and Computed tomography image)
Disease-free survival probability
(Computed tomography image)
Disease-free survival probability
(Patient background)
Disease-free survival probability
(Patient background, Computed tomography findings and Computed tomography image)
Disease-free survival probability
(Patient background and Computed tomography findings)
3

## Slide 4
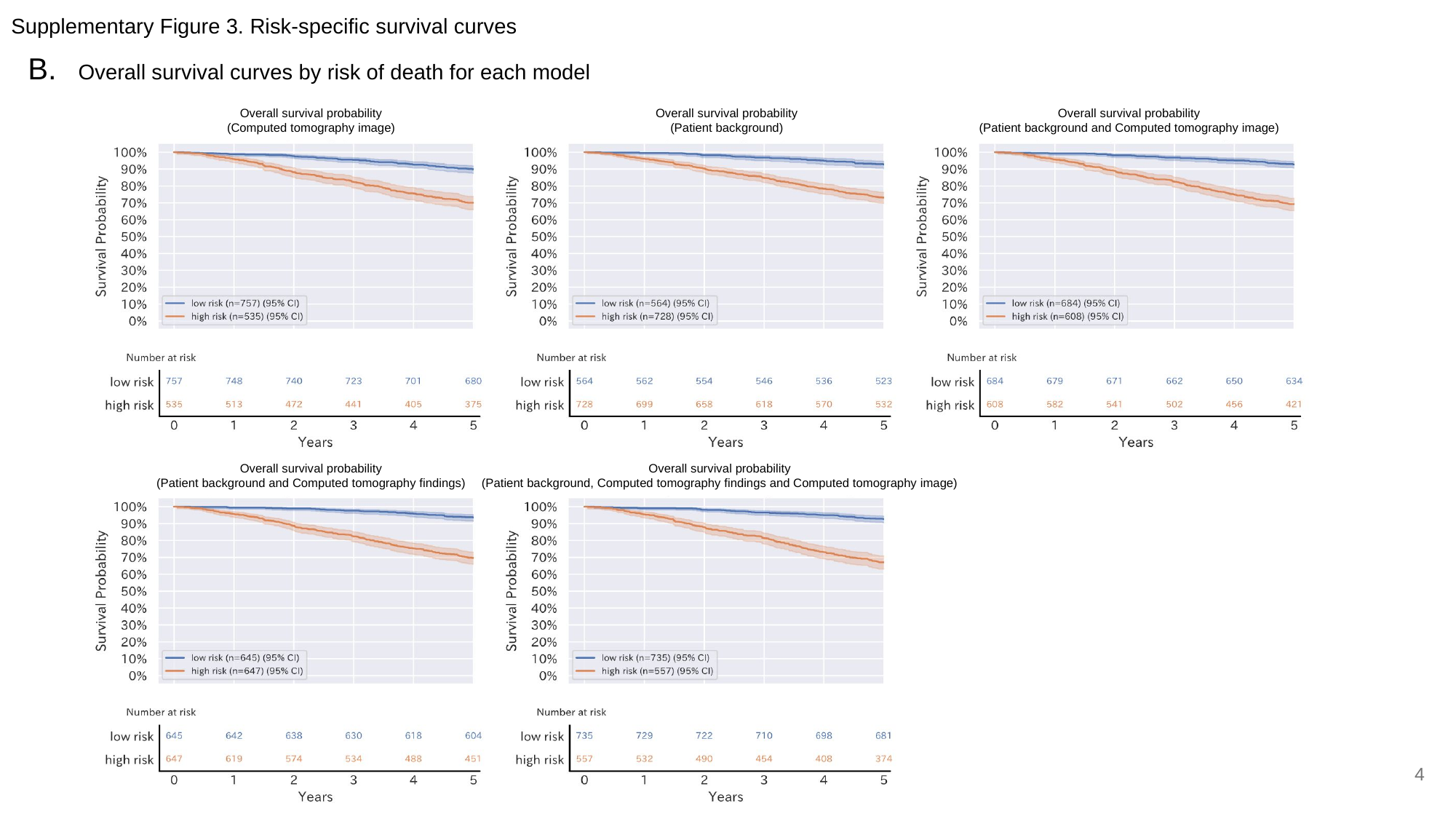

Supplementary Figure 3. Risk-specific survival curves
B.
Overall survival curves by risk of death for each model
Overall survival probability
(Computed tomography image)
Overall survival probability
(Patient background)
Overall survival probability
(Patient background and Computed tomography image)
Overall survival probability
(Patient background and Computed tomography findings)
Overall survival probability
(Patient background, Computed tomography findings and Computed tomography image)
4

## Slide 5
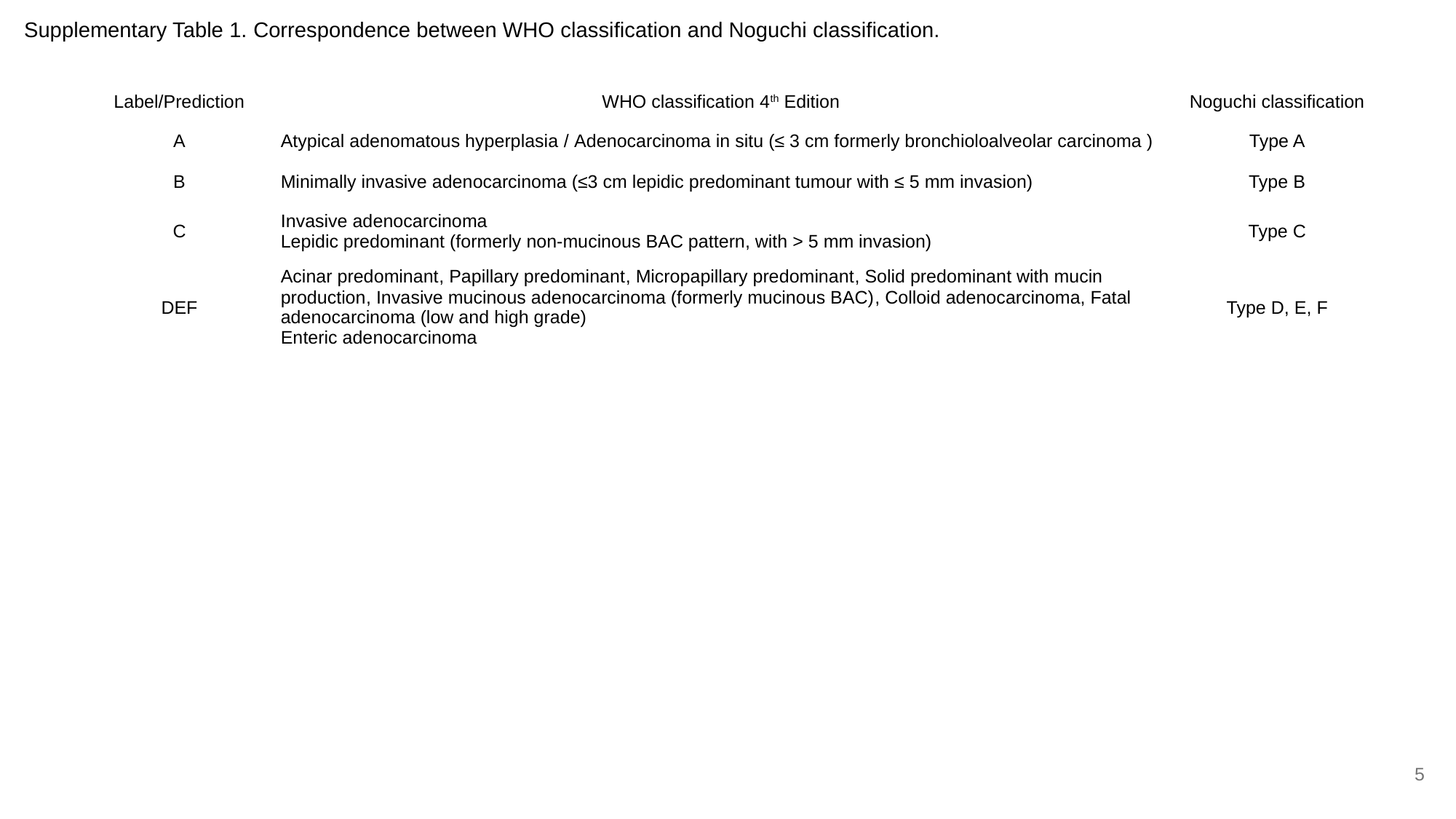

Supplementary Table 1. Correspondence between WHO classification and Noguchi classification.
| Label/Prediction | WHO classification 4th Edition | Noguchi classification |
| --- | --- | --- |
| A | Atypical adenomatous hyperplasia / Adenocarcinoma in situ (≤ 3 cm formerly bronchioloalveolar carcinoma ) | Type A |
| B | Minimally invasive adenocarcinoma (≤3 cm lepidic predominant tumour with ≤ 5 mm invasion) | Type B |
| C | Invasive adenocarcinoma Lepidic predominant (formerly non-mucinous BAC pattern, with > 5 mm invasion) | Type C |
| DEF | Acinar predominant, Papillary predominant, Micropapillary predominant, Solid predominant with mucin production, Invasive mucinous adenocarcinoma (formerly mucinous BAC), Colloid adenocarcinoma, Fatal adenocarcinoma (low and high grade) Enteric adenocarcinoma | Type D, E, F |
5

## Slide 6
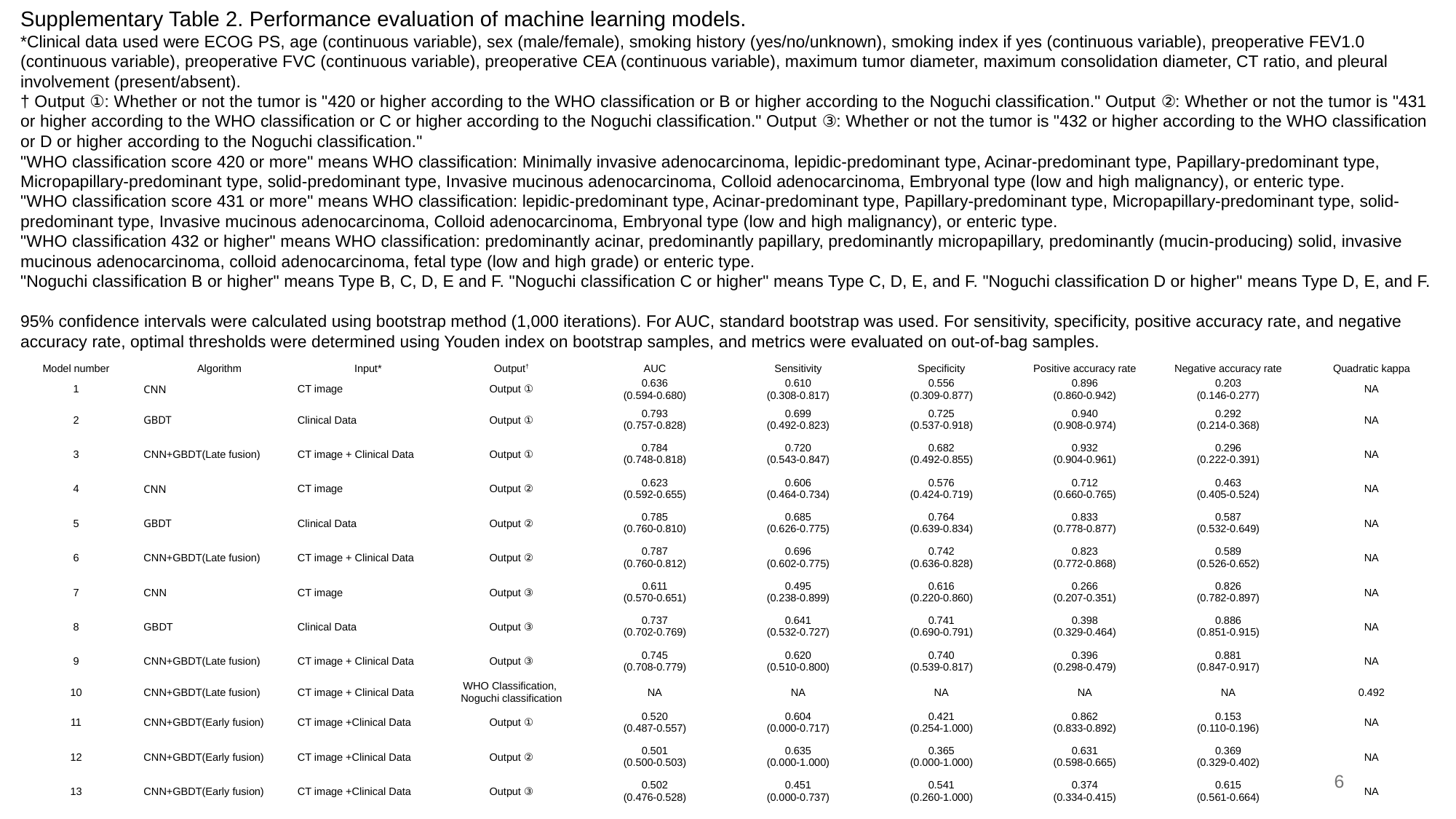

Supplementary Table 2. Performance evaluation of machine learning models.
*Clinical data used were ECOG PS, age (continuous variable), sex (male/female), smoking history (yes/no/unknown), smoking index if yes (continuous variable), preoperative FEV1.0 (continuous variable), preoperative FVC (continuous variable), preoperative CEA (continuous variable), maximum tumor diameter, maximum consolidation diameter, CT ratio, and pleural involvement (present/absent).
† Output ①: Whether or not the tumor is "420 or higher according to the WHO classification or B or higher according to the Noguchi classification." Output ②: Whether or not the tumor is "431 or higher according to the WHO classification or C or higher according to the Noguchi classification." Output ③: Whether or not the tumor is "432 or higher according to the WHO classification or D or higher according to the Noguchi classification."
"WHO classification score 420 or more" means WHO classification: Minimally invasive adenocarcinoma, lepidic-predominant type, Acinar-predominant type, Papillary-predominant type, Micropapillary-predominant type, solid-predominant type, Invasive mucinous adenocarcinoma, Colloid adenocarcinoma, Embryonal type (low and high malignancy), or enteric type.
"WHO classification score 431 or more" means WHO classification: lepidic-predominant type, Acinar-predominant type, Papillary-predominant type, Micropapillary-predominant type, solid-predominant type, Invasive mucinous adenocarcinoma, Colloid adenocarcinoma, Embryonal type (low and high malignancy), or enteric type.
"WHO classification 432 or higher" means WHO classification: predominantly acinar, predominantly papillary, predominantly micropapillary, predominantly (mucin-producing) solid, invasive mucinous adenocarcinoma, colloid adenocarcinoma, fetal type (low and high grade) or enteric type.
"Noguchi classification B or higher" means Type B, C, D, E and F. "Noguchi classification C or higher" means Type C, D, E, and F. "Noguchi classification D or higher" means Type D, E, and F.
95% confidence intervals were calculated using bootstrap method (1,000 iterations). For AUC, standard bootstrap was used. For sensitivity, specificity, positive accuracy rate, and negative accuracy rate, optimal thresholds were determined using Youden index on bootstrap samples, and metrics were evaluated on out-of-bag samples.
| Model number | Algorithm | Input\* | Output† | AUC | Sensitivity | Specificity | Positive accuracy rate | Negative accuracy rate | Quadratic kappa |
| --- | --- | --- | --- | --- | --- | --- | --- | --- | --- |
| 1 | CNN | CT image | Output ① | 0.636 (0.594-0.680) | 0.610 (0.308-0.817) | 0.556 (0.309-0.877) | 0.896 (0.860-0.942) | 0.203 (0.146-0.277) | NA |
| 2 | GBDT | Clinical Data | Output ① | 0.793 (0.757-0.828) | 0.699 (0.492-0.823) | 0.725 (0.537-0.918) | 0.940 (0.908-0.974) | 0.292 (0.214-0.368) | NA |
| 3 | CNN+GBDT(Late fusion) | CT image + Clinical Data | Output ① | 0.784 (0.748-0.818) | 0.720 (0.543-0.847) | 0.682 (0.492-0.855) | 0.932 (0.904-0.961) | 0.296 (0.222-0.391) | NA |
| 4 | CNN | CT image | Output ② | 0.623 (0.592-0.655) | 0.606 (0.464-0.734) | 0.576 (0.424-0.719) | 0.712 (0.660-0.765) | 0.463 (0.405-0.524) | NA |
| 5 | GBDT | Clinical Data | Output ② | 0.785 (0.760-0.810) | 0.685 (0.626-0.775) | 0.764 (0.639-0.834) | 0.833 (0.778-0.877) | 0.587 (0.532-0.649) | NA |
| 6 | CNN+GBDT(Late fusion) | CT image + Clinical Data | Output ② | 0.787 (0.760-0.812) | 0.696 (0.602-0.775) | 0.742 (0.636-0.828) | 0.823 (0.772-0.868) | 0.589 (0.526-0.652) | NA |
| 7 | CNN | CT image | Output ③ | 0.611 (0.570-0.651) | 0.495 (0.238-0.899) | 0.616 (0.220-0.860) | 0.266 (0.207-0.351) | 0.826 (0.782-0.897) | NA |
| 8 | GBDT | Clinical Data | Output ③ | 0.737 (0.702-0.769) | 0.641 (0.532-0.727) | 0.741 (0.690-0.791) | 0.398 (0.329-0.464) | 0.886 (0.851-0.915) | NA |
| 9 | CNN+GBDT(Late fusion) | CT image + Clinical Data | Output ③ | 0.745 (0.708-0.779) | 0.620 (0.510-0.800) | 0.740 (0.539-0.817) | 0.396 (0.298-0.479) | 0.881 (0.847-0.917) | NA |
| 10 | CNN+GBDT(Late fusion) | CT image + Clinical Data | WHO Classification, Noguchi classification | NA | NA | NA | NA | NA | 0.492 |
| 11 | CNN+GBDT(Early fusion) | CT image +Clinical Data | Output ① | 0.520 (0.487-0.557) | 0.604 (0.000-0.717) | 0.421 (0.254-1.000) | 0.862 (0.833-0.892) | 0.153 (0.110-0.196) | NA |
| 12 | CNN+GBDT(Early fusion) | CT image +Clinical Data | Output ② | 0.501 (0.500-0.503) | 0.635 (0.000-1.000) | 0.365 (0.000-1.000) | 0.631 (0.598-0.665) | 0.369 (0.329-0.402) | NA |
| 13 | CNN+GBDT(Early fusion) | CT image +Clinical Data | Output ③ | 0.502 (0.476-0.528) | 0.451 (0.000-0.737) | 0.541 (0.260-1.000) | 0.374 (0.334-0.415) | 0.615 (0.561-0.664) | NA |
6

## Slide 7
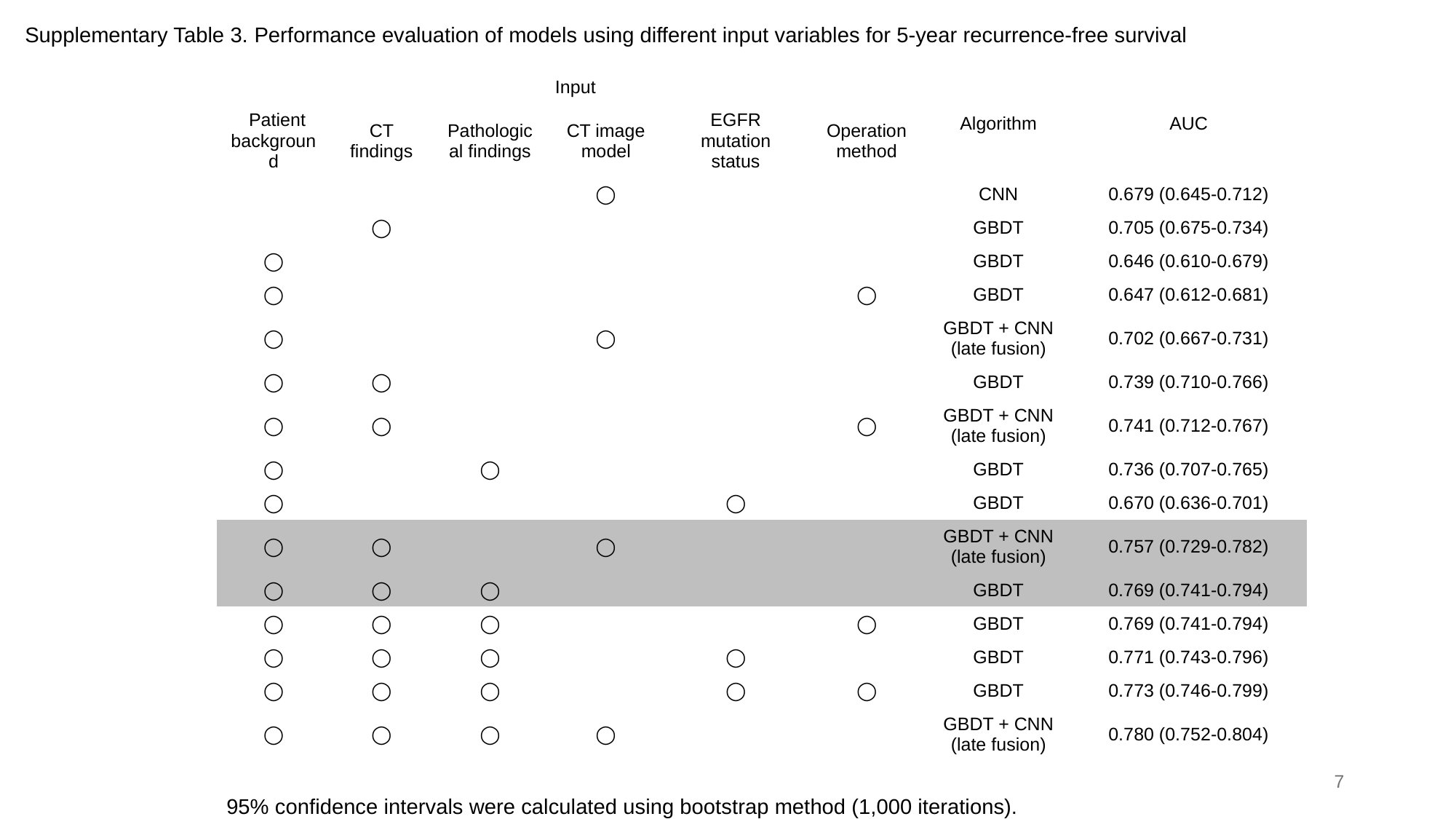

Supplementary Table 3. Performance evaluation of models using different input variables for 5-year recurrence-free survival
| Input | | | | | | Algorithm | AUC |
| --- | --- | --- | --- | --- | --- | --- | --- |
| Patient background | CT findings | Pathological findings | CT image model | EGFR mutation status | Operation method | Algorithm | AUC |
| | | | ◯ | | | CNN | 0.679 (0.645-0.712) |
| | ◯ | | | | | GBDT | 0.705 (0.675-0.734) |
| ◯ | | | | | | GBDT | 0.646 (0.610-0.679) |
| ◯ | | | | | ◯ | GBDT | 0.647 (0.612-0.681) |
| ◯ | | | ◯ | | | GBDT + CNN (late fusion) | 0.702 (0.667-0.731) |
| ◯ | ◯ | | | | | GBDT | 0.739 (0.710-0.766) |
| ◯ | ◯ | | | | ◯ | GBDT + CNN (late fusion) | 0.741 (0.712-0.767) |
| ◯ | | ◯ | | | | GBDT | 0.736 (0.707-0.765) |
| ◯ | | | | ◯ | | GBDT | 0.670 (0.636-0.701) |
| ◯ | ◯ | | ◯ | | | GBDT + CNN (late fusion) | 0.757 (0.729-0.782) |
| ◯ | ◯ | ◯ | | | | GBDT | 0.769 (0.741-0.794) |
| ◯ | ◯ | ◯ | | | ◯ | GBDT | 0.769 (0.741-0.794) |
| ◯ | ◯ | ◯ | | ◯ | | GBDT | 0.771 (0.743-0.796) |
| ◯ | ◯ | ◯ | | ◯ | ◯ | GBDT | 0.773 (0.746-0.799) |
| ◯ | ◯ | ◯ | ◯ | | | GBDT + CNN (late fusion) | 0.780 (0.752-0.804) |
7
95% confidence intervals were calculated using bootstrap method (1,000 iterations).

## Slide 8
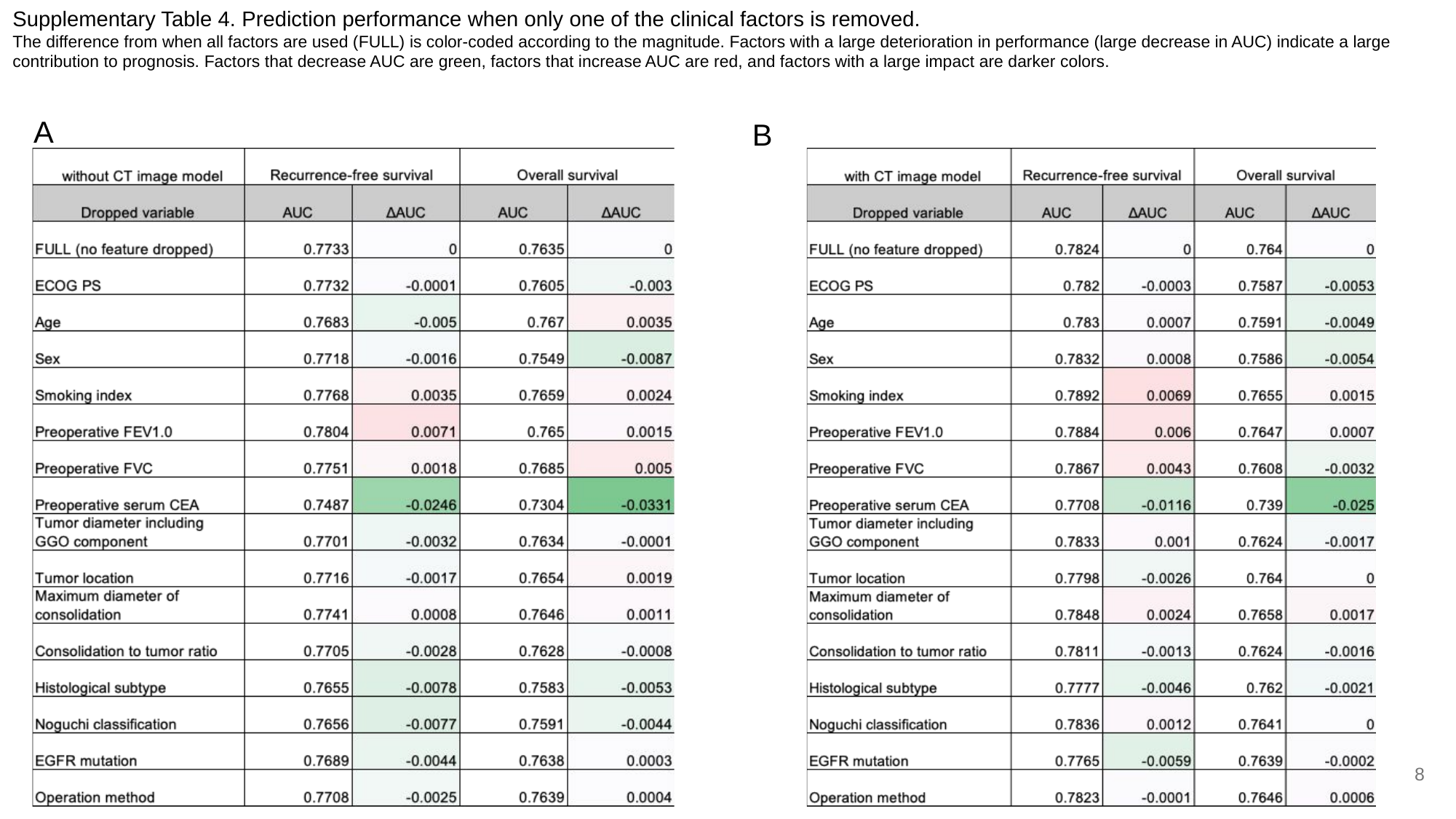

Supplementary Table 4. Prediction performance when only one of the clinical factors is removed.
The difference from when all factors are used (FULL) is color-coded according to the magnitude. Factors with a large deterioration in performance (large decrease in AUC) indicate a large contribution to prognosis. Factors that decrease AUC are green, factors that increase AUC are red, and factors with a large impact are darker colors.
A
B
8
